# Massive subcutaneous abscess: A case report of management and source control

**DOI:** 10.1016/j.ijscr.2024.109638

**Published:** 2024-04-20

**Authors:** Rinta Prasetiyanti, Muhamad Robi'ul Fuadi, Yufi Aulia Azmi, Soetojo Wirjopranoto

**Affiliations:** a Department of Clinical Pathology, Faculty of Medicine, Universitas Airlangga, Dr. Soetomo General Academic Hospital, Surabaya, Indonesia; b Department of Urology, Faculty of Medicine Universitas Airlangga, Universitas Airlangga Academic Hospital, Surabaya, Indonesia; c Department of Health Sciences, University of Groningen, University Medical Center Groningen, Groningen, the Netherlands

**Keywords:** Acute abdomen, Subcutaneous abscess, Bladder repair, Sepsis, Surgery, Case report

## Abstract

**Introduction and importance:**

Postoperative peritoneal infection, a common complication, remains prevalent despite surgical advancements. Acute abdomen necessitates rapid treatment, often presenting with abdominal pain and systemic inflammation. Bladder injuries, potentially leading to sepsis, require immediate surgical intervention.

**Case presentation:**

We report a case of a 60-year-old man who came with the main complaint of feeling full in his stomach for 7 days, accompanied by non-radiating right lower abdominal pain since one day before hospital admission and a lethargy condition. There are complaints of seepage from the stitch marks on the right stomach, such as yellow urine. Laboratory and physical examination showed the patient in sepsis condition. CT Cystography showed a defect of 0.4 cm on the bladder dome, the contrast leakage into extraperitoneal and intraperitoneal, and tunneling to the right abdominal subcutaneous. The patient underwent subcutaneous abscess, bladder repair, and cystostomy. One month after surgery, the patient had normal micturition.

**Clinical discussion:**

Acute abdominal pain is one sign of emergency surgery. It can be caused by infection, inflammation, vascular occlusion, or obstruction. Physical and laboratory examination of the patient showed a sepsis condition. CT Cystography showed the presence of bladder rupture and subcutaneous abscess. The only management is surgical exploration for infection source control.

**Conclusions:**

This case underscores the importance of prompt diagnosis and comprehensive management, involving surgical intervention and targeted antibiotics, for sepsis-related complications post-TURP and bladder repair, necessitating a multidisciplinary approach for optimal outcomes and complication prevention.

## Highlights

• Acute abdominal pain is a very common problem, and it can be possible as an emergency condition.
• Acute abdominal pain may signal emergency surgery due to infection, inflammation, vascular occlusion, or obstruction.
• The only management is surgical exploration for infection source control.
• This case is complex and related to sepsis, which involves multiple surgery procedures.
• Source control surgery is needed to overcome the sepsis condition.

## 1 Introduction

Postoperative peritoneal infection is the most frequent form of intra-abdominal infection, accounting for up to 65 % of all abdominal infections observed in ICU patients [1]. Despite the advancement of surgical technology, postoperative intra-abdominal abscesses still occur after intra-abdominal surgery, especially in diabetes mellitus, obesity [2], and also worsened in immunocompromised persons [1]. This complication may begin with acute abdominal pain is a very common problem and can cause an emergency. The potential causes of an acute abdomen are many and very varied [3]. Acute abdomen requires rapid attention and treatment. Infection, inflammation, vascular occlusion, or obstruction can all result in an acute abdomen. Patients usually present with sudden abdominal pain with associated nausea or vomiting. In general, acute abdominal findings are indications of emergency problems [4]. There are no specific data available, however, abdominal pain accounts for 7 % to 10 % of emergency department visits. CDC data from 1999 to 2008 showed abdominal pain represented 12.5 % of patient presentations and urges. Approximately 30 % of patients suffer from acute renal colic [5]. Patients typically are usually presented to the emergency department with abdominal pain and a systemic inflammatory response, featuring fever, tachycardia, and tachypnea. Abdominal stiffness indicates peritonitis [6].

Bladder injury requires surgical intervention due to the possibility of intra-abdominal sepsis [7]. Sepsis can cause emergencies in these cases and requires appropriate management. This case report explores treatment for acute abdominal caused by subcutaneous abscess after bladder repair. This case report has been reported in line with the SCARE Guideline [8].

## 2 Case presentation

A 60-year-old man came with the main complaint of feeling full in his stomach for 7 days, accompanied by non-radiating right lower abdominal pain. There are complaints of seepage from the stitch marks on the right stomach, such as yellow urine. There was spontaneous smooth urination. Before the complaints occurred, the patient had a history of transurethral resection of the prostate (TURP) and bladder repair with 2 layers of bladder stitching. The bladder rupture was caused by an iatrogenic bladder rupture caused by the TURP procedure at a previous hospital. The first bladder repair was done 11 days before the complaint when admitted to the hospital at the referral hospital. Subcutaneous abscess, possibly from urine leakage from bladder rupture.

**Fig. 1:**
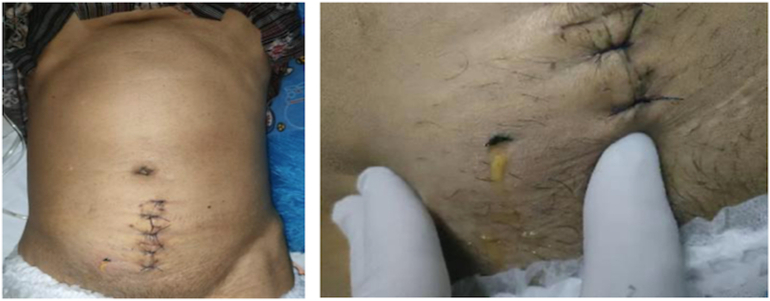
Results of abdominal examination.

The physical examination showed a poor general condition characterized by mild decreased consciousness (14 scores on the Glasgow Coma Scale), anemia, and fever, with a temperature of 38.7 degrees Celsius. Hemodynamic parameters indicated a blood pressure of 90/54 mmHg, a heart rate of 100 bpm, and a respiratory rate of 24 breaths per minute. The abdomen was distended, and the suprapubic showed an empty bladder, post-operative scar (+) and urinary leakage (+) (Fig. 1).

**Fig. 2:**
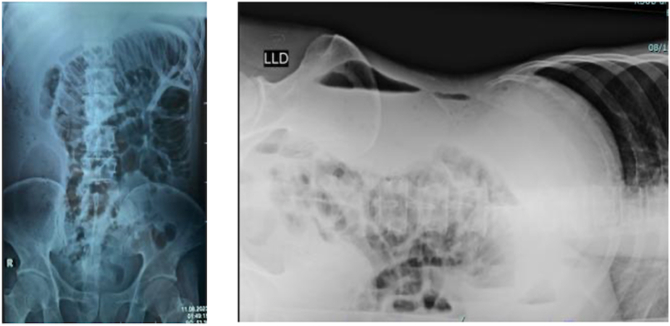
Results of KUB Examination.

The patient underwent laboratory and radiological examinations. Laboratory results showed leukocytosis 20.12 and elevated procalcitonin 3.14. The results of the Kidney Ureter Bladder (KUB) examination showed an oval-shaped opacity, clear boundaries, and regular edges, measuring +/− 6.7 × 11.3 projected at the level of VTh 3 to the sacrum on the right side of the abdomen, which on the lateral photo appeared to form an air-fluid level (+). The Left Lateral Decubitus (LLD) examination showed that the shadow of intestinal gas was only visible and distributed in the left abdominal area. No pathological step ladder was visible. No shadow of free air was visible in the abdominal cavity. (Fig. 2).

**Fig. 3:**
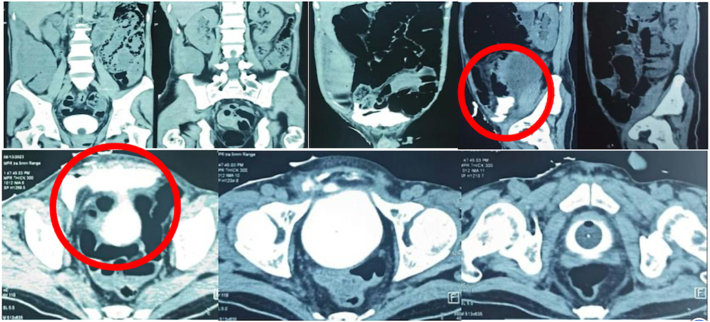
CT Cystography Examination.

CT cystography results showed there was a defect in the right anterior wall of the bladder with a size of +/− 0.4 cm, which caused extravasation of contrast to the extraperitoneal, then filled the tract in *musculus rectus abdominis* to the right side abdominal subcutaneous, and some of the contrast enters through the intraperitoneal filling (Fig. 3).

**Fig. 4:**
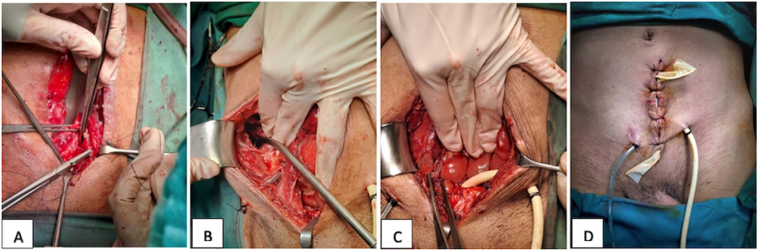
(A) Bladder repair, (B) subcutaneous abscess drainage, (C) open cystostomy.

**Fig. 5:**
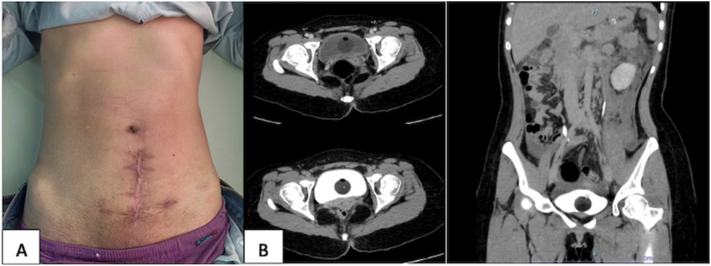
(A) Clinical Picture Post Operation Follow Up (B) CT Cystography Evaluation.

To avoid worsening conditions, the patient got ceftriaxone 1 g/12 h IV and metronidazole 500 mg/ 8 IV hours before surgical exploration. The patient underwent laparotomy exploration, midline infra umbilical incision was performed, and then a bladder rupture was found in the dome area size 10 cm and looked like it split; bladder repair and an open cystostomy were performed (Fig. 4). Also, subcutaneous abscess was found in the right quadrant of the abdomen, then abscess drainage was performed, total 1500 cc of pus was found in the subcutaneous region of the right quadrant of the abdomen. A sample of pus was collected for culture and antibiotic susceptibility testing. A rectal tube drain was placed in the subcutis region in the right quadrant of the abdomen, installing a redon drain in the retzii cavity and placing a subcutis handschoen drain in the midline incision, then primary suturing was performed. The patient was maintained with cystostomy and followed up (Fig. 5). The results of culture and antibiotic sensitivity revealed the growth of *Enterobacter cloacae*, which exhibited sensitivity to the administration of antibiotics Amikacin, Imipenem, and Meropenem. The administration of antibiotics with Ceftriaxone and Metronidazole was continued due to clinical improvement, characterized by the patient's resolution of fever and improved hemodynamic parameters. Additionally, there was an improvement in laboratory parameters, indicated by a decrease in leucocyte count, from 20.12 to 11.47 and procalcitonin levels, from 3.14 to 2.31. The broad-spectrum antibiotics were administered until the patient was discharged from the hospital at 5-day post-operative. At the two-week follow-up, the patient reported no complaints, the surgical wound healed well and no pus discharge was observed from the surgical wound.

## 3 Discussion

This case is a complex related to sepsis. Management also varies for acute abdominal cases. Evaluation of emergency department patients with acute abdominal pain might be challenging. Several factors can conceal presentation, delaying or preventing an accurate diagnosis and resulting in undesirable patient outcomes. In these circumstances, doctors consider several diagnoses, particularly those that are life-threatening conditions and require prompt treatment to reduce morbidity and mortality [9].

In this case, the patient had a TURP and bladder repair history. TURP is a procedure that removes the prostate and is resected by an endoscopic approach. It was the first essential minimally invasive surgery in the modern era. TURP can also be performed to unroof prostate abscesses and unblock the ejaculatory ducts in certain cases of obstructive azoospermia. TURP is a procedure used to treat bladder outlet obstruction caused by prostatic enlargement as well as prostate abscess [10]. The patient, in this case, also underwent bladder repair. Bladder injury needs surgical intervention due to the possibility of intra-abdominal sepsis.

Bladder injuries are classified as intraperitoneal and extraperitoneal injuries. Extraperitoneal bladder injuries are commonly associated with pelvic fractures, whereas intraperitoneal bladder injuries result from high-energy impacts on an overly distended bladder. Extraperitoneal bladder injuries are treated with indwelling catheters, whereas intraperitoneal and complex bladder injuries are surgically repaired. Non-operative treatment for uncomplicated bladder injuries without urethral injury are treated with an indwelling catheter for 10 to 14 days along with antibiotic prophylaxis [7].

The patient had developed sepsis in 10 days after the initial surgical procedures, TURP and bladder repair, indicating the presence of healthcare-associated infections (HAIs) potentially attributed to surgical wound infection. Sepsis is described as life-threatening organ dysfunction induced by an abnormal host response to infection. Organ dysfunction is defined as an acute rise in two or more points on the Sequential Organ Failure Assessment (SOFA) score [11]. In this patient, a SOFA score of 4 was obtained based on parameters including a mean arterial pressure (MAP) below 70, a Glasgow Coma Scale (GCS) score of 14, and a bilirubin level of 4.6. Meanwhile, a qSOFA score of 3 points was observed based on the parameters of level of consciousness, respiratory rate and systolic blood pressure, indicating organ dysfunction due to sepsis-related to abdominal wall abscess.

In this case, the patient underwent abscess drainage. An abscess is a localized accumulation of purulent fluid that can significantly impact a patient's care and clinical outcome. Sometimes, this is a minor event that may be effectively treated with antibiotics only. Nevertheless, abscess formation cannot infrequently be a life-threatening event if it leads to sepsis, a severe spectrum of systemic disease caused by hematogenous spread of infection, and a substantial source of morbidity and mortality [12]. Incision and drainage (I&D) is commonly utilized in a variety of healthcare settings, involving emergency departments and outpatient clinics. This is the standard treatment for skin and soft tissue abscesses, regardless of additional antibiotics. In most cases, skin abscesses can usually be clinically diagnosed using only a physical examination. The typical features of an abscess are induration, erythema, tenderness on palpation, and fluctuation [13]. The patient also underwent an open cystotomy and bladder repair. The rectus fascia is opened to access the preperitoneal space [14].

This patient was given empirical antibiotics with Ceftriaxone and Metronidazole before surgey. Based on guidelines from the Surgical Infection Society (SIS), the recommended antibiotics regimen for empirical therapy are antibiotics that are active against the typical gram-negative Enterobacteriaceae, gram-positive cocci, and obligate anaerobes. One of the recommended combination regimens is Ceftriaxone and Metronidazole [15]. Despite the pus culture results of this patient showing growth of *Enterobacter cloacae* resistant to Ceftriaxone based on antibiotic susceptibility test, Ceftriaxone administration was maintained as the combination with Metronidazole revealed improvement in clinical and laboratory parameters.

The emergence of abscess in this case is attributed to *Enterobacter cloacae* infection, as evidenced by pus culture examination. *Enterobacter cloacae* is commensal microflora in the human intestinal tracts. *Enterobacter* spp. Including *Enterobacter cloacae* can lead to urinary tract infections (UTIs), bacteremia, lower respiratory tract infections, surgical site infections, and colonization of intravascular devices among other things [16]. This microorganism is among the *Enterobacter* spp. most implicated in nosocomial infections in the last decade, with numerous publications documenting the antibiotic resistance features of this microorganism [17]. The pathogenic mechanisms and contributing factors associated with diseases related to abscess formation with *E. cloacae* remain poorly understood [17]. The formation of an abscess in the abdominal wall may potentially be attributed to urinary leakage resulting from vesicocutaneous fistula, characterized by the discharge of urine from the post-operative wound [18], as observed in this patient. Fourteen days after surgery, CT cystography was performed to evaluate the complex bladder rupture, and the result was normal.

## 4 Conclusion

This case highlights the complexity of managing sepsis-related complications following TURP and bladder repair procedures. Prompt diagnosis and appropriate management, including surgical intervention and targeted antibiotic therapy, are crucial for achieving favorable outcomes. Despite the challenges posed by antibiotic-resistant pathogens such as *Enterobacter cloacae*, a multidisciplinary approach involving urologists, infectious disease specialists, and surgeons is essential for the effective management of such cases and the prevention of further complications.

## Ethical approval

Ethical approval has been acquired in this study by Health Research Ethics Committee of Dr. Soetomo General-Academic Hospital, Surabaya, Indonesia.

## Funding

This research did not receive any specific grant from funding agencies in the public, commercial, or not-for-profit sectors.

## CRediT authorship contribution statement

Rinta Prasetiyanti: Conceptualization, Methodology, Data Curation, Investigation, Writing-Original draft preparation

Muhamad Robi’ul Fuadi: Conceptualization, Data Curation, Writing-Original draft preparation

Yufi Aulia Azmi: Data Curation, Writing original draft-Reviewing, and Editing

Soetojo Wirjopranoto: Writing iriginal draft, Reviewing, Supervision, Validation

## Guarantor

Mohamad Robi'ul Fuadi, Soetojo Wirjopranoto.

## Declaration of competing interest

The authors declare that there is no conflict of interest.

## Data Availability

No data was used for the research described in the article.
